# Therapeutic Efficacy of Platelet-Rich Fibrin in Delayed Replantation of an Avulsed Tooth: A Clinical Case Report on Management and Outcome Assessment

**DOI:** 10.7759/cureus.86029

**Published:** 2025-06-14

**Authors:** Akansha Tilokani, Yash Sinha, Prasanti Pradhan, Sonali Bansal, Aditi Gupta

**Affiliations:** 1 Department of Conservative Dentistry and Endodontics, Kalinga Institute of Dental Sciences, Bhubaneswar, IND; 2 Department of Conservative Dentistry and Endodontics, Indira Gandhi Institute of Medical Sciences, Patna, Patna, IND; 3 Department of Pediatric and Preventive Dentistry, Kalinga Institute of Dental Sciences, Bhubaneswar, IND

**Keywords:** avulsion, delayed replantation, dental trauma, extraoral root canal treatment, platelet-rich fibrin (prf)

## Abstract

This case report describes the successful management of a delayed replantation of an avulsed maxillary central incisor with prolonged extraoral dry time (>60 minutes). A 24-year-old patient presented with a dehydrated avulsed tooth, which underwent extraoral root canal treatment and replantation using platelet-rich fibrin (PRF) to enhance healing. The tooth was splinted for four weeks, and the patient was followed up for two years. The replanted tooth remained asymptomatic, stable, and showed no signs of resorption or infection. This case highlights the importance of a multidisciplinary approach, integrating advanced biological and clinical techniques, in achieving favourable outcomes in traumatic dental injuries.

## Introduction

Avulsion injuries account for approximately 0.5% to 16% of all dental traumatic injuries [1]. Permanent maxillary central incisors are most frequently avulsed due to trauma; however, the prognosis for these teeth is often suboptimal, as evidenced by various studies [2]. Replantation is typically the preferred course of action; however, immediate replantation is not always possible. Effective emergency care and a well-structured treatment plan are crucial for a favourable outcome. There are instances where replantation may not be suitable, such as in cases of advanced caries, severe periodontal disease, significant cognitive impairment requiring sedation, or serious medical conditions like immunosuppression or cardiac disorders. Each case must be carefully evaluated.

Replanting an avulsed tooth offers the possibility of preservation, but the likelihood of long-term survival is limited. Choosing not to replant a tooth is an irreversible decision, making it essential to attempt preservation whenever feasible. The efficacy of replantation is mostly contingent upon the state of the periodontal ligament (PDL), the developmental stage of the tooth's root, and the duration of time the tooth has been extricated from the oral cavity. Replantation within the first five minutes yields the highest chances of successful healing through PDL regeneration, provided that the innermost cell layers along the root surface remain viable [3].

Replanted teeth are generally functional for an average of five years but are often ultimately lost due to replacement resorption or inflammatory root resorption. Studies have shown that teeth left outside the oral environment for over two hours have a significantly high risk of external resorption, approximately 95%. Nevertheless, avulsed teeth with viable PDL cells can be successfully replanted and function for several years with proper management [4].

## Case presentation

On March 14, 2023, a 24-year-old patient reported to the Department of Conservative Dentistry and Endodontics with a chief complaint of a displaced upper front tooth following trauma six hours prior. The tooth avulsion was caused by slipping on a hand pump. The patient did not experience any loss of consciousness or bleeding from the ears or nose. The avulsed tooth was presented in a dehydrated state without proper preservation. A fracture of the incisal surface with involvement of dentin and enamel, along with dried-out PDL tissue, was seen.

**Figure 1:**
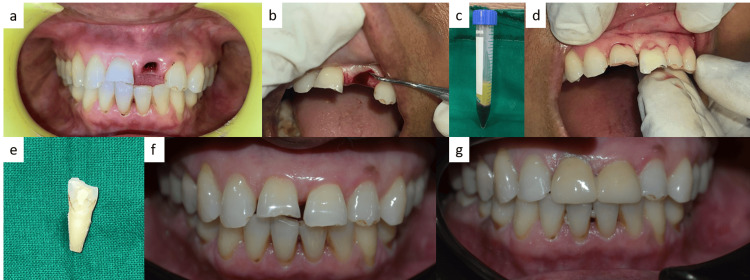
(a) Preoperative image; (b) Curettage of the socket; (c) Preparation of platelet-rich fibrin (PRF); (d) Placement of the tooth and PRF; (e) Root canal-treated tooth; (f) One-year follow-up; (g) Two-year follow-up.

Extraoral inspection was unrevealing for facial swelling, jaw deviation, deflection, limited mouth opening, or step deformity. An empty socket filled with clots was seen intraorally in tooth 21. The patient presented with a Class 1 molar alignment and an overjet measuring 3 mm. Radiographs indicated a healthy socket with no evidence of alveolar fracture, and the teeth in relation to the injured area were normally mobile (Figure 1a).

**Figure 2:**
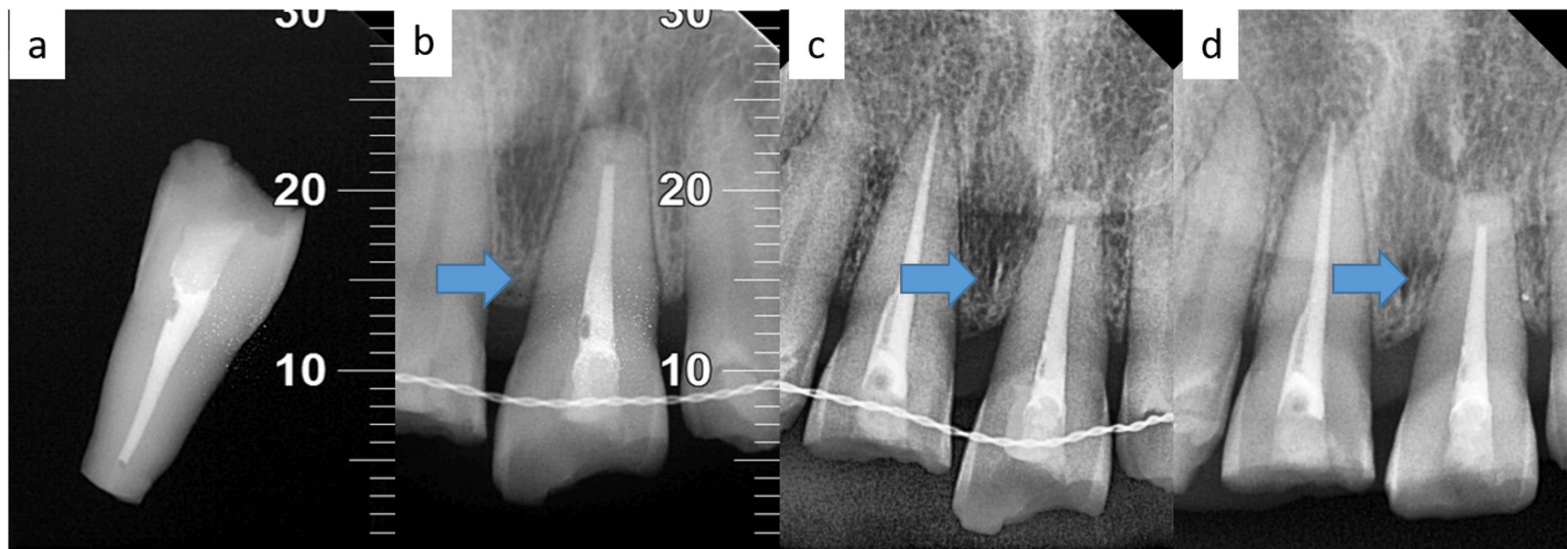
(a) Root canal treatment of tooth 21; (b) Radiograph taken after replantation of tooth, and splinting was done in relation to tooth 21 (blue arrow: avulsed tooth); (c) One-year follow-up (blue arrow: avulsed tooth); (d) Two-year follow-up (blue arrow: avulsed tooth). The markings in Figures 2a-2b serve as anatomical reference points, facilitating the accurate repositioning and verification of the avulsed tooth in relation to the adjacent dentition, thereby ensuring optimal occlusal and spatial relationships.

Replantation of the avulsed tooth was proposed, and consent was received from the patient for the procedure. The tooth was immersed in a solution containing 1 mg of doxycycline in 20 mL of normal saline for 20 minutes, subsequently washed, and dried. The PDL fibers were extracted with a Gracy curette (Hu-Friedy Mfg. Co., LLC, Chicago, IL, USA) and soft pumice prophylaxis. The extraoral dry time of more than 60 minutes dictated extraoral root canal treatment (RCT). The teeth underwent biomechanical preparation (BMP) using size 10-55 Kerr files (Mani Inc., Tochigi, Japan), access opening, and working length determination. Using the lateral condensation approach, gutta-percha was used to obturate the root canal (Figure 1e and Figure 2a).

The socket was cleaned and curetted under local anesthesia (lignocaine 2%, LOX® 2%, Neon Laboratories Ltd., Mumbai, India) with 5% normal saline (Figure 1b). The patient’s blood was used to prepare platelet-rich fibrin (PRF), and it was centrifuged at 2700 rpm for 12 minutes before placing it into the socket (Figure 1c). The tooth was replanted, hand compressed into its original place, and the presence of the tooth was confirmed radiographically (Figure 1d). The tooth was splinted from canine to canine using a 0.4-mm stainless steel wire and composite resin (Tetric N-Ceram, Ivoclar, Schaan, Liechtenstein) (Figure 2b). The patient was prescribed antibiotics along with analgesics for seven days. A soft diet and proper oral hygiene were recommended for two weeks. The patient was also referred for a tetanus booster evaluation. The splint was left in place for four weeks.

The follow-up of one year and two years was uneventful, and the replanted tooth was not symptomatic with no mobility, swelling, or sinus tract development (Figures 1f-1g and Figure 2c). The neighbouring teeth were normal clinically and radiographically. Later, prosthetic rehabilitation was completed (Figure 1g).

Both inflammatory and replacement resorption can occur within three to six months of replantation. Unless detected within two years, resorption reduces the risk significantly. For the aforementioned case, two years after the event, the tooth was asymptomatic, firm, resorption-free, and infection-free radiographically, and with the lamina dura intact, indicative of healthy healing (Figure 2d).

## Discussion

Management of avulsed teeth is a challenging scenario in dental trauma, requiring timely and appropriate intervention to maximize the chances of success. This case report highlights a structured approach to the management of an avulsed tooth with prolonged extraoral dry time, achieving favourable outcomes through meticulous adherence to established protocols [1].

Avulsion injuries involve displacement of a tooth from its socket, often leading to significant damage to the PDL and potential contamination of the root surface [5]. The success of replantation depends on several factors, including the extraoral time, storage medium, and promptness of treatment. In this case, the avulsed tooth was stored in dry conditions for over 60 minutes, leading to desiccation of the PDL tissues. Studies have shown that extended dry time significantly compromises the viability of PDL cells, necessitating alternative approaches to optimise the chances of success [6].

Given the extended extraoral dry time, performing RCT outside the oral cavity was a prudent choice. Endodontic treatment of an avulsed tooth before reimplantation increases the probability of retention and mitigates replacement resorption [7]. The avulsed tooth was soaked in doxycycline for 20 minutes before extraoral filling and replantation. This was done to utilize its antimicrobial properties and to condition the root by exposing collagen fibers on the root cementum, thereby creating a surface conducive to the reattachment of periodontal collagen fibers.

Additionally, PRF, derived from the patient’s own blood, was used in the socket to enhance healing. PRF acts as a reservoir of growth factors, stimulating angiogenesis and osteogenesis, which are critical for the repair of periodontal and alveolar structures. The inclusion of PRF in the treatment protocol reflects the integration of biologically advanced techniques into routine dental trauma management [8].

Studies have demonstrated that splinting techniques allow physiological tooth movement during the healing process, and when used for a minimal duration, reduce the risk of ankylosis. In this case, splinting was performed using a composite and an arch wire. This approach is advantageous as it exerts significantly less stress on the injured area compared to other methods, minimizing the forces applied [9].

Inflammatory and replacement resorption typically become evident within two to six months following reimplantation [10]. However, if resorption is not observed within two years, the likelihood of its occurrence significantly diminishes. In the presented case, after two years, the tooth remained asymptomatic, stable, and showed no radiographic signs of resorption or infection. The presence of an intact lamina dura indicated successful and favourable healing.

## Conclusions

The successful replantation of the avulsed tooth, despite prolonged extraoral dry time, demonstrates the importance of a multidisciplinary approach in managing traumatic dental injuries. By integrating advanced biological and clinical techniques, such as PRF and meticulous splinting, favourable outcomes can be achieved. The two-year follow-up revealed that the tooth remained asymptomatic, stable, and free from resorption or infection. This case highlights the significance of prompt and proper management, preserving both function and aesthetics for the patient. A well-structured treatment plan and adherence to established protocols can optimize the chances of success in similar cases.

## References

[1] Fouad AF, Abbott PV, Tsilingaridis G (2020). International Association of Dental Traumatology guidelines for the management of traumatic dental injuries: 2. Avulsion of permanent teeth. Dent Traumatol.

[2] Barrett EJ, Kenny DJ (1997). Survival of avulsed permanent maxillary incisors in children following delayed replantation. Endod Dent Traumatol.

[3] Wang G, Wang C, Qin M (2019). A retrospective study of survival of 196 replanted permanent teeth in children. Dent Traumatol.

[4] Jain S, Agarwal V, Gupta AK, Prabhakar P (2012). Replantation of immature avulsed teeth with prolonged extraoral dry storage: a case report. Int J Clin Pediatr Dent.

[5] Weine PS (1996). Endodontic Therapy. 5th Edition. https://www.scirp.org/reference/referencespapers?referenceid=2196550.

[6] Harris A, Reshmi J, George S, Issac JS (2014). Delayed reimplantation: a case report. J Int Oral Health.

[7] Ize-Iyamu IN, Saheeb B (2013). Reimplantation of avulsed dry permanent teeth after three days: a report of two cases. Niger J Clin Pract.

[8] Yang Y, Liu YL, Jia LN, Wang JJ, Zhang M (2023). Rescuing "hopeless" avulsed teeth using autologous platelet-rich fibrin following delayed reimplantation: two case reports. World J Clin Cases.

[9] Finucane D, Kinirons MJ (2003). External inflammatory and replacement resorption of luxated, and avulsed replanted permanent incisors: a review and case presentation. Dent Traumatol.

[10] Puri S, Tripathi S, Pandya M, Trivedi P (2011). Reimplantation of avulsed teeth after dry storage for one week. Int J Clin Dent Sci.

